# Supplementary material for: Networks of genetic similarity reveal non-neutral processes shape strain structure in Plasmodium falciparum
Source: Nat Commun. 2018 May 8;9:1817. doi: 10.1038/s41467-018-04219-3 (PMC5940794; doi:10.1038/s41467-018-04219-3)
Supplement: Supplementary file 1 — Supplementary Information [file 41467_2018_4219_MOESM1_ESM.pdf]

Supplementary Information

He et al. Networks of genetic similarity reveal non-neutral processes shape strain structure in *Plasmodium falciparum*

Contents:

Supplementary Table 1-4
Supplementary Fig. 1-9
Supplementary Note 1
Supplementary References

Supplementary Table 1 | List of network properties used for network characterization and classification.

No	Network Property	Description
	**Metrics of clustering coefficient**	The degree to which nodes in a graph tend to cluster together
**1**	Average local clustering coefficient	Ratio of the triangles connected to a node and the triples centered on the node (undirected, unweighted networks)[1], averaged across nodes.
**2**	Average weighted local clustering coefficient	Local clustering coefficient in an undirected, weighted network[2]
**3**	Global clustering coefficient	The ratio of the triangles and the connected triples in the network[3]
	**Metrics related to degree/strength**	Number of edges per node
**4**	Graph density	The ratio of the number of edges and the number of possible edges, excluding self loops[3]
**5**	Proportion of nodes with degree 0	
**6**	Proportion of nodes with degree 1	
**7**	Average degree	Node degree, averaged across nodes
**8**	Assortativity	Pearson correlation coefficient of the degrees at either ends of an edge[4]
**9**	Average strength	Sum of edge weights incident to a node, averaged across nodes.
**10**	Straightness (Power law test)	Pearson coefficient of a power-law degree distribution[5]
**11**	Average measurement of the heterogeneity of the network	Entropy of the degree distribution[5]
**12**	Number of components relative to network size	
**13**	Average component size	
**14**	Entropy of component size distribution	
	**Geodesic distance**	Shortest path between two vertices
**15**	Central point dominance	Average difference in betweenness centrality between the most central node and all others[6]
**16**	Average eccentricity	Average of the longest of all shortest paths from a node to all other nodes[7]
**17**	Diameter	Length of the longest shortest path between any pairs of nodes
**18**	Mean diameter of all components	The diameter of each network component, averaged across components
**19**	Reciprocal of global efficiency	Harmonic mean of the geodesic distances[5,8]
**20**	Average closeness centrality	Number of hops required to access every other node from a given node[6], averaged across nodes.
	**Graph motifs proportions**	Percentage of each motif among all 3-node motifs within the network[9]
**21**	A->B<-C, the binary in-tree.	
**22**	A->B->C, the directed line.	
**23**	A<->B<-C.	
**24**	A<-B->C, the binary out-tree.	
**25**	A->B<-C, A->C.	
**26**	A<-B->C, A<->C.	
**27**	A<->B->C.	
**28**	A<->B<->C.	
**29**	A->B->C, A<->C.	
**30**	A->B<-C, A<->C.	
**31**	A->B<->C, A<->C.	
**32**	A<->B<->C, A<->C, the complete graph.	
	**In and out edges**	
**33**	Reciprocity	Proportion of mutual connections
**34**	In-out correlation	Correlation between the in-degree and out-degree of nodes, for all nodes.
	**Modules and F_{ST}**	Community detected with Newman-Girvan algorithm[10]
	Q	Maximum modularity[10], not used for classification
	Maximum F_{ST}	diversity within community compared with between communitites[11,12], not used for classification

Supplementary Table 2 | Network classification accuracy as a function of gene pool size and percentile of retained edges. Accuracy is calculated as the sum of true positives and true negatives divided by the number of total test cases. Gene pool size: 12000-24000 (high); 1200-2400 (low).

			Accuracy		
Season	Gene pool size	Top percentile of strong edges	Immune selection	Generalized immunity	Complete neutrality
Non-seasonal	high	80.00	0.990	0.720	0.720
		60.00	1.000	0.695	0.695
		40.00	1.000	0.720	0.720
		20.00	1.000	0.700	0.700
		15.00	1.000	0.705	0.705
		10.00	1.000	0.710	0.710
	low	80.00	0.900	0.790	0.800
		60.00	0.885	0.820	0.815
		40.00	0.880	0.775	0.765
		20.00	0.879	0.794	0.794
		15.00	0.899	0.779	0.799
		10.00	0.953	0.844	0.839
Seasonal	high	62.50	0.940	0.999	0.939
		50.00	0.933	0.997	0.930
		37.50	0.953	0.996	0.949
		25.00	0.961	0.994	0.956
		20.00	0.989	0.996	0.984
		15.00	0.984	0.997	0.981
		10.00	0.981	0.996	0.983
		2.50	0.991	0.997	0.989

Supplementary Table 3 | Characteristics of immune selection (balancing selection) captured by different network metrics compared with traditional approaches from community ecology and population genetics. Network properties are more informative of population structure compared to diversity measures commonly used in ecology (see Supplementary results and discussion; Supplementary Fig. 9). Moreover, these properties are more appropriate for describing similarity patterns in the presence of frequent recombination than population genetics measures assuming the existence of a 'tree'-like phylogeny. The table summarizes general patterns that arise from balancing selection and includes a specific pattern relevant for gene families. The correspondence between common tests or indices in ecology and population genetics is shown, together with their predictions relative to those of neutral scenarios (in parentheses). Network metrics are for the most part explained in Methods and in the Supplementary table 1. Remaining ones are referred to specific figures or references in this table.

Patterns	Community ecology	Population genetics*	Network metrics
**Increased diversity around targets of selection**	Shannon diversity *H* (higher)[13]	Hudson–Kreitman–Agaudé test (much higher polymorphism than divergence)[14]	Greater number of components (see Fig. 1 and Extended Data Fig. 2)
**Evenly sized niches (ecology); Excess of common polymorphisms (evolution)**	Evenness (higher)[15]	Fu and Li's *F*[16], Tajima's *D*[17] (positive)	Negative relationships between frequency and relatedness (see text and Extended Data Fig. 4)
	Abundance distribution (skewed toward intermediate-abundance)[18]	Allele frequency spectrum (skewed toward intermediate-frequency alleles)[19]	Even component/community sizes (see Fig. 1 and Extended Data Fig. 2)
**Persistence of niches (ecology); Shared variants across species (evolution)**		F_{ST} among species compared to neutral sites (lower)	Persistence of strain modules (not tested in this paper)
		Persistence of similar gene variants across species	
**Shared diversity across location**	Beta diversity across locations (lower)[20]	F_{ST} among locations compared to neutral genes (lower)	Module identities have less correlation with locations than neutral genes (not tested in this paper)
**Limiting similarity (ecology); Linkage disequilibrium among genes (evolution)**	d/w (niche divergence over niche width; wider)[21]	Long Range Haplotype test (longer haplotype than expected)[22]	Modularity (see Fig. 1 and Extended Data Fig. 2)[10]
	Pairwise type sharing (low)[23]	Integrated Extended Haplotype Homozygosity (negative, long haplotypes associated with derived alleles)[24]	Module F_{ST} (see Methods for definition and references, Fig. 1 and Extended Data Fig. 2)
	k-means dispersion[25]		Transitivity
	Ripley's K function[26]		Higher proportion of complete graph in motif compositions (see Fig. 1 and Extended Data Fig. 2)
**High within-genome diversity of gene families**			High reciprocity (see Fig. 1 and Extended Data Fig. 2)

*** Tests listed under population genetics are adapted from Fijarczyk and Babik[27], where more tests and references are reviewed and discussed.**

Supplementary Table 4 | Description of parameters and selected ranges used in the extended *var* evolution model.

Symbol	Type	Description	Values
H	Integer	Host population size	10,000
G	Integer	Size of *var* gene pool	1,200 – 24,000
g	Integer	Size of each repertoire (i.e., number of *var* genes per repertoire)	10 - 60
l	Integer	Number of epitopes per *var* gene	2, 5
n_i	Integer	Number of allele variants per epitope i	120 – 2,400
b	Rate	Biting event rate per host	0.05-0.5
p	Probability	Transmission probability	0.5
ρ	Rate	Mitotic recombination rate per gene	1.8e-07/day
μ	Rate	Mutation rate per epitope per gene	1.42e-08/day
w	Rate	Rate of specific epitope immunity wane per day	0.001
m	Rate	Migration rate of new genomes	1 genome per day
D	Rate	Duration of a naïve infection in the immune selection scenario	3 months to 2 years

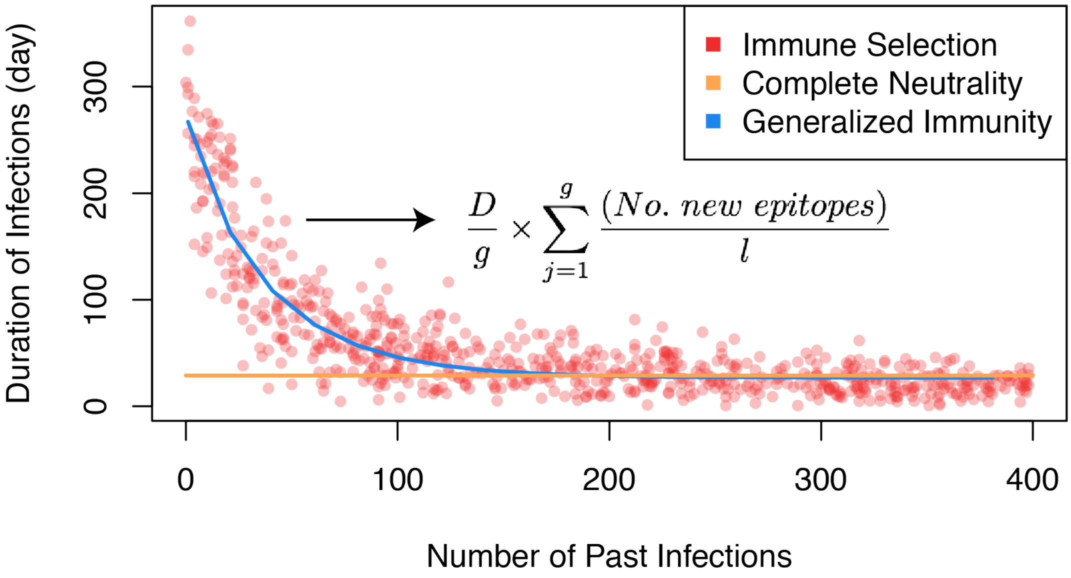

Supplementary Figure 1| Duration of infection under the three models. For a given set of parameters, the 'immune selection' model (red dots) was run first and the duration of infection and the number of past infections were recorded. Duration of an infection in a specific host is a function of the number of past infections (as shown in the equation), given that previously seen epitopes are not expressed. For the purpose of meaningful comparison, we set infection duration in the complete neutrality model to be the average duration across all infections computed from runs of the 'immune selection' model (yellow line). For the 'generalized immunity' model (blue line), we set the duration to be the average duration for a given number of past infections computed from the 'immune selection' model.

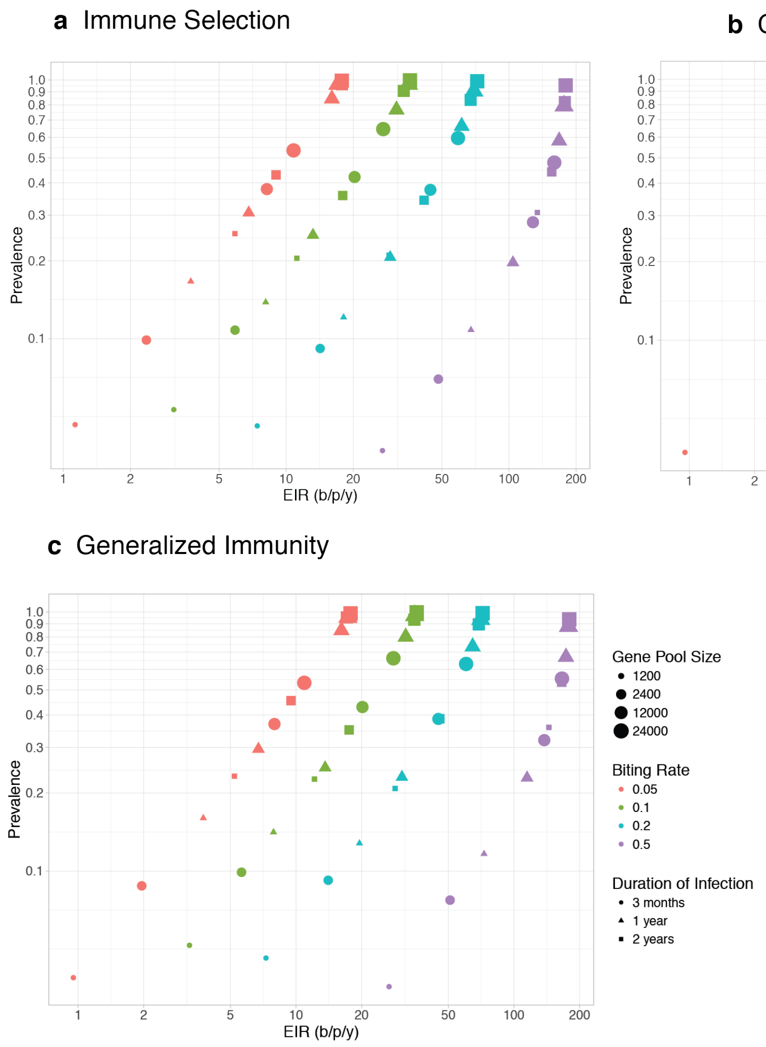

Supplementary Figure 2| Relationship between the entomological Inoculation Rate (EIR) and prevalence (log scale) in the simulation given different combinations of parameters. EIR is measured as the number of infectious bites per person per year.

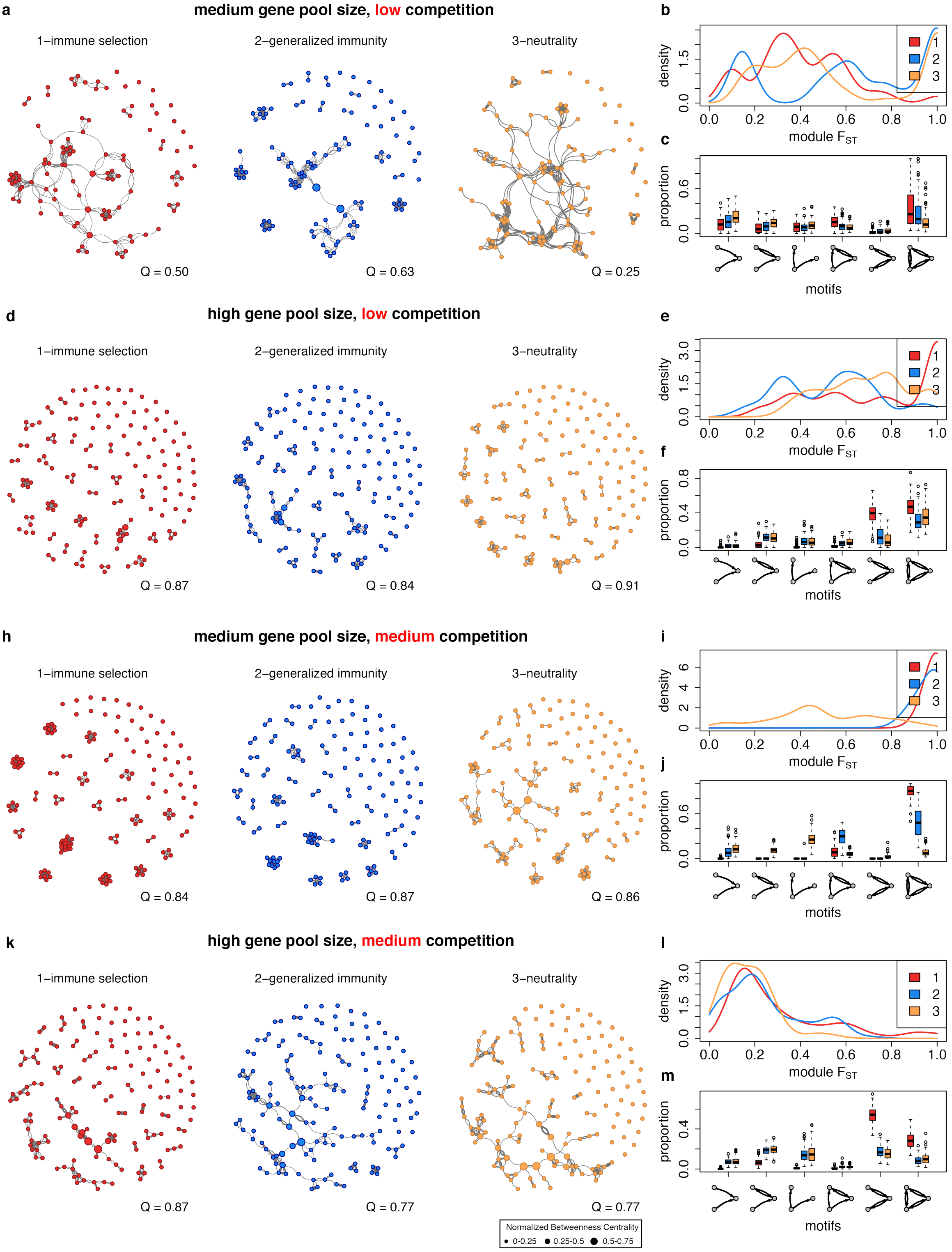

Supplementary Figure 3 | Repertoire similarity networks and representative network metrics across scenarios for different diversity regimes generated with low and medium duration of naïve infection and transmission rates. First and third panels, medium gene pool size (G = 1,200) and second and fourth panels, high gene pool size (G = 24,000). **a, d**, **h**, **k**, comparisons of repertoire similarity networks of 150 randomly sampled parasite *var* repertoires from one time point under the three scenarios. Only the top 1% of the strongly connected links are drawn and used in the analyses, with the thickness of the edges representing the relative strength of connections within the network (see Supplementary Fig.

3 for distribution of edge weights and degrees). Within the largest component of each network, the size of each node indicates its normalized betweenness centrality. The value of maximum modularity Q is calculated using edge-betweenness and shown at the lower right corner of each network. **b, e**, **i**, **l**, pairwise module F_{ST} distributions. **c, f**, **j**, **m**, proportion of occurrence of 3-node graph motifs for the three models.

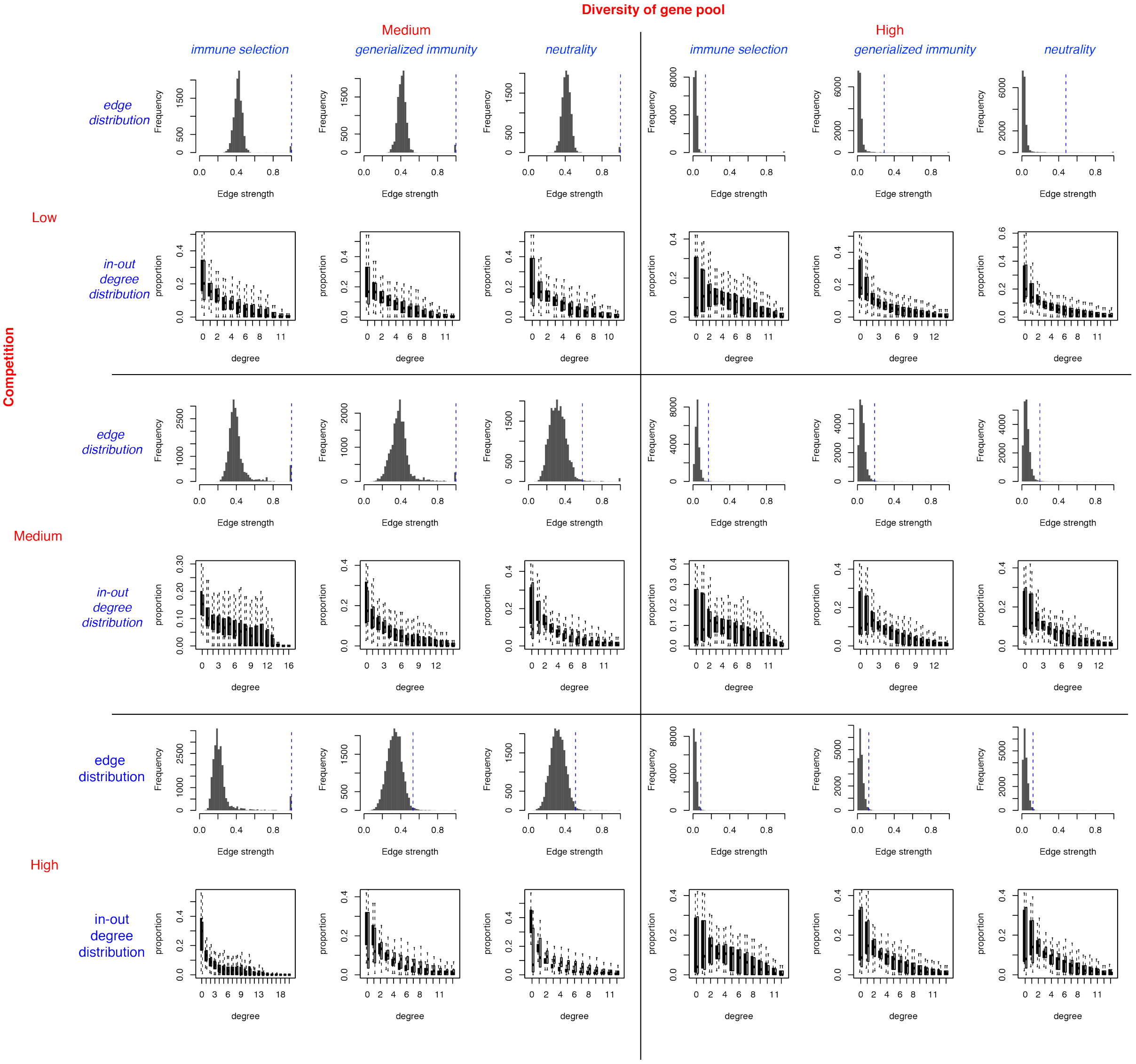

Supplementary Figure 4 | Edge weight distributions and in-out degree distributions under different gene pool diversity and competition regimes. Distributions are ordered from medium diversity (left) to high diversity (right), and low competition (upper) to high competition (lower). Under each regime, scenarios are shown for immune selection (left), generalized immunity (middle) and neutrality (right). The blue dotted lines in the edge distribution plots show the repertoire similarity cutoff (for the top 1% of edge weights used in building the networks). In and out edges refer to the direction the edge is pointing respective to the focal node. In-degree measures how many other nodes the focal node receives connections from, with these connections above cut-off. Out-degree measures how many other nodes the focal node sends connections to, with these connections above cut-off. See the asymmetric measure defined in Supplementary Fig. 8. Bar plots of in- and out-degree are shown across 100 networks that are generated under these same regimes (respectively in black and grey).

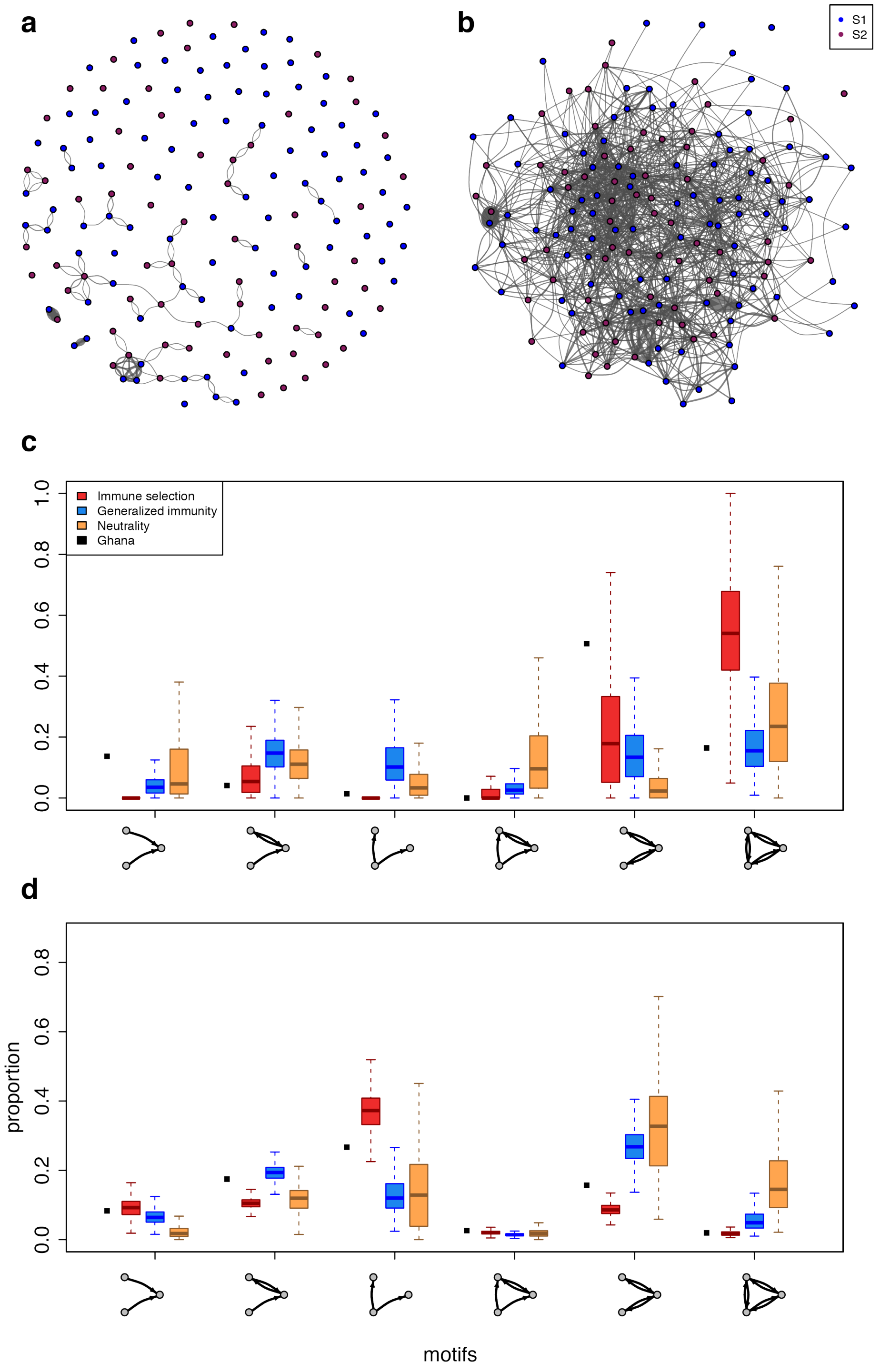

Supplementary Figure 5 | Strain similarity network of var upsB/C DBLα types in the Ghana samples show high proportion of reciprocally connected motifs. a, The top 1% of edges (i.e., $S_{ij} > 0.0755$) is shown in the network. **b**, The top 10% of edges (i.e., $S_{ij} > 0.0455$) is shown in the network and used in the discriminant analysis of principal components in Fig. 5. The color of each node represents the season in which the isolate was sampled (S1: October 2012, end of wet season; S2: beginning of June 2013, end of dry season). **c**, **d**, Proportion of motifs among the simulated seasonal networks under the three scenarios compared with those of the empirical data from Ghana using top 1% and 10% of edge weights, respectively.

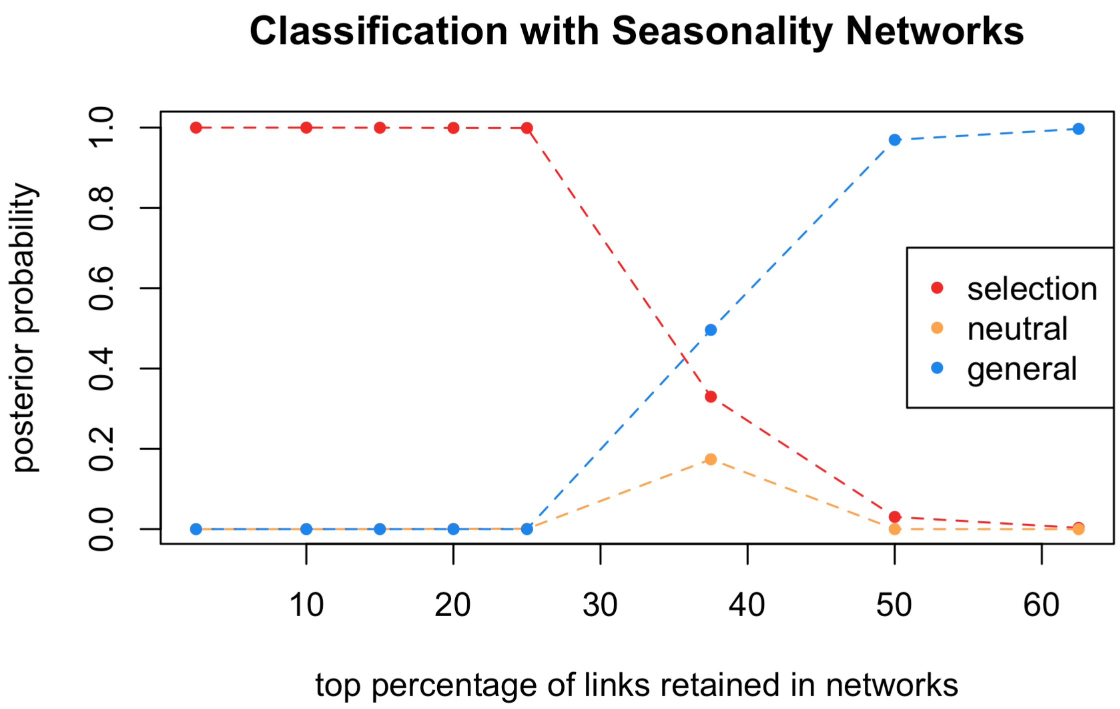

Supplementary Figure 6 | Network classification of data from Bongo District, Ghana with percentage of top edges retained in the networks. When classification is performed with the top 1-25% of edge weights of the empirical network, the algorithm assigns the empirical data as most likely to be generated from the selection scenario. When including weaker edges, the classification identifies generalized immunity as the most likely scenario. Because these weaker edges (representing the lower 75% of the strength of edges in the empirical network) correspond to the sharing of only one gene between pairs of repertoires, results are largely driven by common *var* types and as such cannot reveal structure emerging due to competition for hosts. We therefore consider these weaker edges as reflecting more of the noise than the signal from underlying processes in the dynamics, and used a cutoff within the higher top percentile for the empirical analyses. Note that for each percentage of links, the posterior probabilities sum to 1.

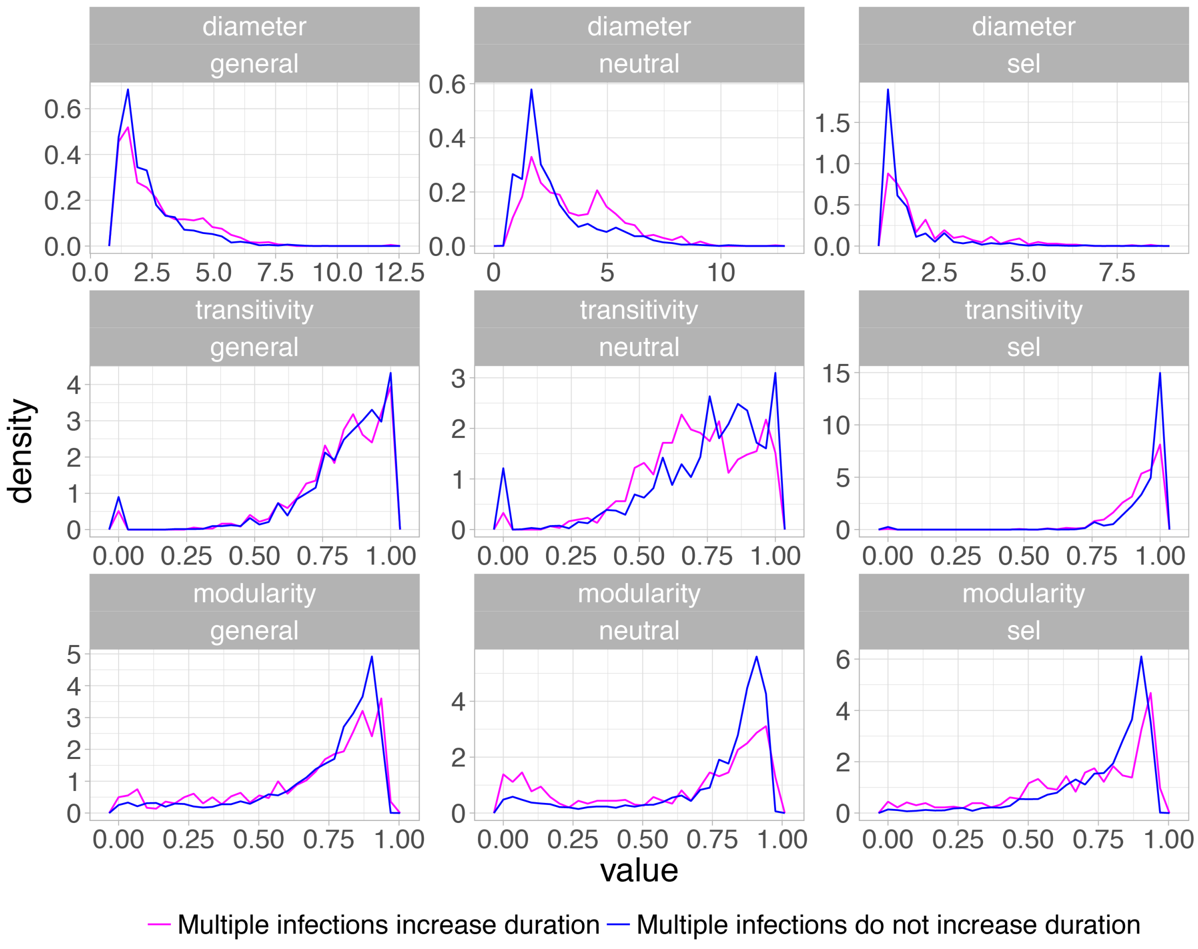

Supplementary Figure 7| Network properties under two different assumptions of within-host dynamics are qualitatively similar. In the main results, we presented models that assume no cost associated with multiplicity of infection (MOI); that is, the duration of infection is not correlated with the number of genomes a host is infected with (blue line). Because there is some evidence for such a positive correlation[28,29], we also explored another version of within-host dynamics, in which the duration of infection increases as a function of MOI as [Duration =D/(MOI^-0.5)]. This mimics the observation that each strain dominates at a different time during the infection due to expression of a different *var*, which prolongs the overall duration of infection for all strains. We compare here three network properties (diameter, transitivity and maximum modularity) across all three models to illustrate that the two scenarios of within-host dynamics produce qualitatively similar network structures. Parameter ranges: *b*~[0.05,0.2], *g*~[10,20], *G*~[500,10000], *l*~[2,5].

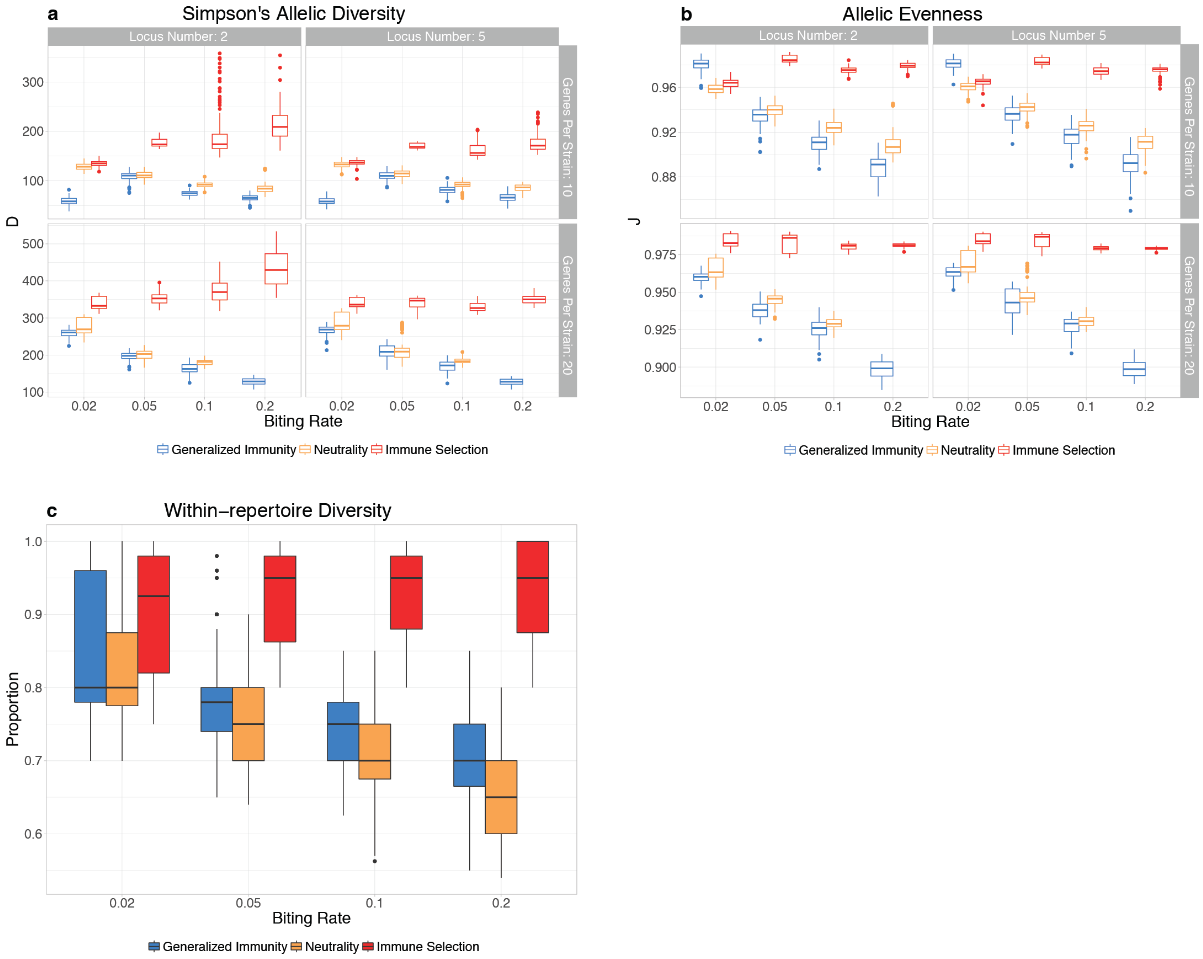

Supplementary Figure 8 | Comparisons of allelic diversity. The comparisons are across immune selection (red), generalized immunity (blue) and neutral (yellow) scenarios. Simpson's allelic diversity (**a**) and allelic evenness *J* (**b**) are compared across biting rates. The number of epitopes per *var* gene (columns) vs. the number of *var* genes per genome (rows). Within repertoire diversity (**c**) is presented as the proportion of unique alleles divided by the length of the genome. Values are combined for all parameter combinations for a given biting rate.

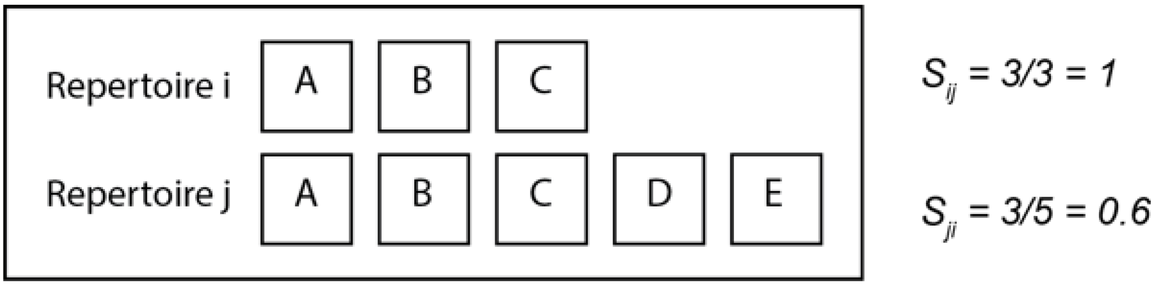

Supplementary Figure 9 | Illustration of the similarity index for edge strength in network construction. We use a directional network because of the asymmetric competition resulting from different numbers of unique variants in a repertoire. In the example, strain *i* has 3 unique alleles, while strain *j* has 5. Together they share 3 alleles. Therefore, strain *i* can be substituted by strain *j* completely, while strain *j* can only be substituted by strain *i* partially. Therefore, strain *j* will have a prolonged expression in a host which is immune to strain *i*, while strain *i* will not be able to cause infection in a host which is immune to strain *j*.

Supplementary Note 1

Selection signature based on standard ecological diversity measures

We investigated whether standard ecological diversity measures can be used to differentiate selection signatures. As expected from standard genetic signatures of frequency-dependent selection, the parasite *var* gene population has higher and more even epitope (allelic) diversities in the selection than in the neutral models for the same parameter ranges (Supplementary Fig. 8a, b). Diversity patterns under generalized immunity, although different from those under complete neutrality, nevertheless resemble those of complete neutrality more than those of immune selection. What is more unique to the system as a result of within-strain competition is a higher within-genome diversity than that of the null models (also see Buckee and Recker[30] on the evolution of multi-domains in gene structures) (Supplementary Fig. 8c). Except for the allelic diversity indices, most of the other diversity indices (such as beta diversity, genetic or repertoire diversity) do not show clear trends differentiating the underlying processes. Because these differences are relative, a given value of these indices would not provide information about underlying processes. In this sense, they are un-informative and would require comparisons across endemicity gradients to provide evidence for non-neutrality in empirical systems.

Supplementary References

1. Watts, D. J. & Strogatz, S. H. Collective dynamics of 'small-world' networks. *Nature* **393,** 440–442 (1998).
2. Barrat, A., Barthélemy, M., Pastor-Satorras, R. & Vespignani, A. The architecture of complex weighted networks. *Proc. Natl. Acad. Sci. U. S. A.* **101,** 3747–3752 (2004).
3. Wasserman, S. & Faust, K. *Social Network Analysis: Methods and Applications*. (Cambridge University Press, 1994).
4. Newman, M. E. J. Assortative Mixing in Networks. *Phys. Rev. Lett.* **89,** 208701 (2002).
5. Costa, L. da F., Rodrigues, F. A., Travieso, G. & Villas Boas, P. R. Characterization of complex networks: A survey of measurements. *Adv. Phys.* **56,** 167–242 (2007).
6. Freeman, L. C. A Set of Measures of Centrality Based on Betweenness. *Sociometry* **40,** 35–41 (1977).
7. Harary, F. *Graph theory*. (Addison-Wesley, 1994).
8. Latora, V. & Marchiori, M. Efficient Behavior of Small-World Networks. *Phys. Rev. Lett.* **87,** 198701 (2001).
9. Cordella, L. P., Foggia, P., Sansone, C. & Vento, M. An improved algorithm for matching large graphs. in *In: 3rd IAPR-TC15 Workshop on Graph-based Representations in Pattern Recognition, Cuen* 149–159 (2001).
10. Newman, M. E. J. & Girvan, M. Finding and evaluating community structure in networks. *Phys. Rev. E* **69,** 026113 (2004).
11. Takahata, N. & Satta, Y. Footprints of intragenic recombination at HLA loci. *Immunogenetics* **47,** 430–441 (1998).
12. Charlesworth, D. Balancing Selection and Its Effects on Sequences in Nearby Genome Regions. *PLOS Genet.* **2,** e64 (2006).

13. Shannon, C. E. A Mathematical Theory of Communication. *SIGMOBILE Mob Comput Commun Rev* **5,** 3–55 (2001).
14. Hudson, R. R., Kreitman, M. & Aguadé, M. A Test of Neutral Molecular Evolution Based on Nucleotide Data. *Genetics* **116,** 153–159 (1987).
15. Pielou, E. C. The measurement of diversity in different types of biological collections. *J. Theor. Biol.* **13,** 131–144 (1966).
16. Fu, Y. X. & Li, W. H. Statistical tests of neutrality of mutations. *Genetics* **133,** 693–709 (1993).
17. Tajima, F. Statistical Method for Testing the Neutral Mutation Hypothesis by DNA Polymorphism. *Genetics* **123,** 585–595 (1989).
18. Alonso, D. & McKane, A. J. Sampling Hubbell's neutral theory of biodiversity. *Ecol. Lett.* **7,** 901–910 (2004).
19. DeGiorgio, M., Lohmueller, K. E. & Nielsen, R. A Model-Based Approach for Identifying Signatures of Ancient Balancing Selection in Genetic Data. *PLOS Genet.* **10,** e1004561 (2014).
20. Koleff, P., Gaston, K. J. & Lennon, J. J. Measuring beta diversity for presence–absence data. *J. Anim. Ecol.* **72,** 367–382 (2003).
21. Macarthur, R. & Levins, R. The Limiting Similarity, Convergence, and Divergence of Coexisting Species. *Am. Nat.* **101,** 377–385 (1967).
22. Sabeti, P. C. *et al.* Detecting recent positive selection in the human genome from haplotype structure. *Nature* **419,** 832–837 (2002).
23. Barry, A. E. *et al.* Population Genomics of the Immune Evasion (var) Genes of Plasmodium falciparum. *PLOS Pathog.* **3,** e34 (2007).
24. Voight, B. F., Kudaravalli, S., Wen, X. & Pritchard, J. K. A Map of Recent Positive Selection in the Human Genome. *PLOS Biol.* **4,** e72 (2006).

25. MacQueen, J. B. Some Methods for classification and Analysis of Multivariate Observations. *5th Berkeley Symp. Math. Stat. Probab.* **1,** 281–297 (1967).

26. Ripley, B. D. *Spatial Statistics*. (Wiley, New York, 1981).

27. Fijarczyk, A. & Babik, W. Detecting balancing selection in genomes: limits and prospects. *Mol. Ecol.* **24,** 3529–3545 (2015).

28. Nassir, E. *et al.* Impact of genetic complexity on longevity and gametocytogenesis of Plasmodium falciparum during the dry and transmission-free season of eastern Sudan. *Int. J. Parasitol.* **35,** 49–55 (2005).

29. Bruce, M. C. *et al.* Genetic diversity and dynamics of Plasmodium falciparum and P. vivax populations in multiply infected children with asymptomatic malaria infections in Papua New Guinea. *Parasitology* **121,** 257–272 (2000).

30. Buckee, C. O. & Recker, M. Evolution of the Multi-Domain Structures of Virulence Genes in the Human Malaria Parasite, Plasmodium falciparum. *PLOS Comput. Biol.* **8,** e1002451 (2012).
