# Supplementary material for: Networks of genetic similarity reveal non-neutral processes shape strain structure in Plasmodium falciparum
Source: Nat Commun. 2018 May 8;9:1817. doi: 10.1038/s41467-018-04219-3 (PMC5940794; doi:10.1038/s41467-018-04219-3)
Supplement: Supplementary file 3 — Description of Additional Supplementary Files [file 41467_2018_4219_MOESM3_ESM.pdf]

Description of Additional Supplementary Files

File Name: Supplementary Data 1
Description: Gene composition of Ghana isolates used in the repertoire analysis. Column names are unique identifiers for upsB/upsC *var* DBLα tags. Row names indicate unique isolates. Values in the matrix refer to the number of copies of the given *var* gene sequenced in the isolate. See Methods for details of the var DBLα sequence analysis.
